# Pigmented (melanotic) diffuse neurofibroma of the back in neurofibromatosis type 1

**DOI:** 10.3205/iprs000124

**Published:** 2018-08-03

**Authors:** Reinhard E. Friedrich, Christian Hagel

**Affiliations:** 1 Department of Oral and Craniomaxillofacial Surgery, Eppendorf University Hospital, University of Hamburg, Germany; 2 Institute of Neuropathology, Eppendorf University Hospital, University of Hamburg, Hamburg, Germany

**Keywords:** neurofibromatosis type 1, melanotic neurofibroma, pigmented neurofibroma, diffuse neurofibroma

## Abstract

Neurofibromatosis type 1 (NF1) is a tumor predisposition disease. Multiple neurofibromas are among the characteristic tumors of NF1. The report describes the diagnosis and treatment of a large spinal neurofibroma in a NF1 patient. The tumor showed a striking pigmentation and was diagnosed as pigmented (melanotic) neurofibroma. The distinction between this rare tumor variant and other pigmented tumors, especially malignant melanoma, is of primary importance.

## Zusammenfassung

Die Neurofibromatose Typ 1 (NF1) ist eine Tumor-Prädispositionserkrankung. Multiple Neurofibrome zählen zu den charakteristischen Tumoren der NF1. Der Bericht beschreibt Diagnose und Behandlung eines ausgedehnten Neurofibroms des Rückens bei einem NF1 Patienten. Intraoperativ zeigte sich eine auffällige Pigmentierung des Tumors, der histologisch als pigmentiertes (melanotisches) Neurofibrom eingeordnet wurde. Die Unterscheidung dieser seltenen Variante des Neurofibroms von anderen pigmentbildenden Tumoren, insbesondere dem malignen Melanom, ist von vorrangiger Bedeutung.

## Introduction

Neurofibromatosis type 1 (NF1) is an autosomal dominant tumor suppressor gene disorder [1]. The diagnosis is established by typical clinical findings in the majority of cases [2]. Minimal requirements have been defined for the diagnosis of NF1. The clinical diagnosis is based on presence of two of the following [2]:

1. Six or more café au lait macules over 5 mm in diameter in prepubertal individuals and over 15 mm in diameter in postpubertal individuals
2. Two or more neurofibromas of any type or one plexiform neurofibroma
3. Freckling in the axillary or inguinal regions
4. Two or more Lisch nodules (iris hamartomas)
5. Optic glioma
6. A distinctive osseous lesion such as sphenoid dysplasia or thinning of long bone cortex, with or without pseudarthrosis
7. First-degree relative (parent, sibling, or offspring) with NF1 by the above criteria

The hallmark of NF1 are benign tumors of the peripheral nervous system termed neurofibroma [3]. The name of the tumor entity refers to a neuronal component comprising transformed Schwann cells and a non-neoplastic fibrous component that includes fibroblasts. Loss of function of the NF1 gene in Schwann cells as a result of mutations in both alleles or one mutated allele plus loss of heterozygosity are considered to be the cause of neurofibroma formation [4]. While the typical cutaneous neurofibroma, which often occur in large numbers, characteristically is observed after puberty, plexiform neurofibroma (PNF) are thought to develop during the embryonic or perinatal period. According to recent findings, the frequency of PNF in NF1 is about 30% [5].

In very rare cases PNF can be pigmented [6], [7], [8] and may look like malignant melanoma intraoperatively. Further, malignant neurofibroma (malignant peripheral nerve sheath tumor, MPNST) may show pigmentation rendering it difficult to distinguish the tumor from a malignant melanoma [9]. Finally, the pigmentation disorder in NF1 may give rise to a malignant melanoma [10].

Schwann cells and melanocytes are derived from the neural crest [11]. Mutations of the NF1 gene in melanocytes have been described in NF1 patients [12], [13]. Ultrastructurally, differences in pigmentation exist between melanin in the skin of NF1 patients and samples of the skin of healthy individuals [14]. Here we report on a patient with NF1 who had developed a large tumor and showed pigmentation both of the covering skin and inside the tumor.

## Case description

This 18-year-old male patient presented at the Oral and Craniomaxillofacial Surgery Clinic to consider surgical treatment options for reducing an enlarged tumor of the back. The patient had more than six café au lait spots on the trunk and extremities, axial and inguinal freckling and several cutaneous tumors that were slightly raised above the level of the skin. The patient had no physical discomfort, no motor or sensitive deficits. The patient stated that he had been operated 2 years earlier on a tumor of the back in another hospital. More detailed information was not available. Despite this previous treatment of the tumor, the remaining tumor mass disturbed him both physically and in his self-perception. The patient stated that the tumor had been growing again since the first operation.

**Figure 1:**
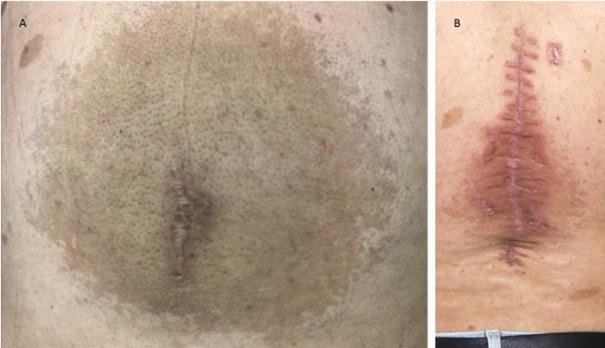
A: Photograph of the lesion of the back prior to surgical intervention. Scar in the midline indicates possible biopsy of previous approach. The tumor is roughly marked by the extension of the circular nevus covering the entire lower back. B: Photograph of the back following debulking procedure and completion of wound healing.

On the back there was a tumorous protrusion of the intact skin with a maximum above the spine, which extended from the lower thoracic region close to the edge of the pelvis (Figure 1 A (Fig. 1)). The tumor was clearly prominent under tight-fitting clothing. The skin in this area was darker pigmented throughout the lumbar region and showed hirsutism. The tumor was insensitive to touch and pressure, showed no fluctuation on palpation, and the covering skin moved with the tumor.

**Figure 2:**
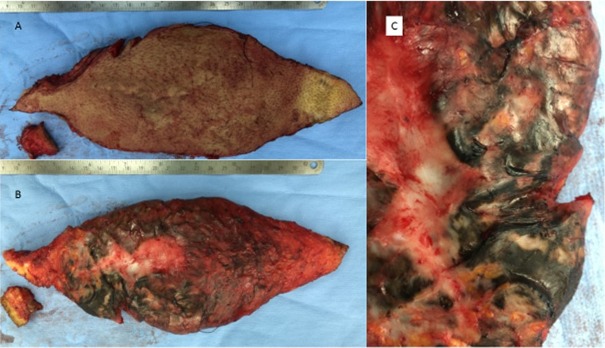
A: Photograph of the resection specimen with hyperpigmented skin. B: Photograph of the resection specimen with the upturned resection surface. Note the stripe-like almost black hyperpigmentations within the lesion. C: Detail of photograph shown in B illustrating strip-like and hyperpigmented parts of the lesion that are irregularly distributed within the tumor.

B-scan ultrasound revealed an inhomogeneous mass with focal, partly string-like reflections inside the space occupying lesion. Borders were poorly defined and the tumor mass reached to the spinous processes of the spine. The tumor appeared as solid mass and contained no cavities suggestive of necrosis. The tumor was resected in general anesthesia. When the lesion was exposed, a black pigmentation became apparent, which was partially arranged in a stripe-like pattern and frayed at the edges (Figure 2 (Fig. 2)). The tumor was resected and the contour of the back reshaped. Despite dense suturing of the wound margins a hematoma developed, which was emptied. Secondary wound healing took 21 days and led to a stable healed wound (Figure 1 B (Fig. 1)). There was no movement restriction of the patient after the wound had healed.

Upon neuropathological investigation a spindle-shaped, 22x9x2 cm^3^ large skin sample with centrally located 6 cm long scar was seen. Cutting the skin exposed white and slightly greasy tissue on both sides of the scar with spotty brown-black pigmentation.

Histological examination revealed a diffusely grown neoplasia of medium to high cellular density in the subepidermal connective tissue, consisting of roundish and oblong cells with delicate cytoplasmic extensions and slightly pleomorphic, small, round-oval, sometimes comma-shaped nuclei. The cells showed different degrees of pigmentation. Repeatedly, pseudo-Meissner corpuscles were observed. There was no evidence of mitoses and no Turnbull-positive hemosiderin pigment was detected. Immunohistochemistry demonstrated labeling of the tumor cells with antibodies against S100-protein and melan-A and to a lesser extent also with antibodies against HMB45. The Ki-67-proliferation index was less than 3%.

**Figure 3:**
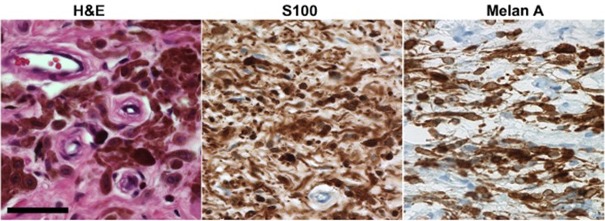
Histology of melanotic neurofibroma depicting clustered and scattered pigmented cells in H&E stain. Tumor cells react with antibodies against S100-protein and melan-A, note the typical wavy contours of the cells in the immunohistochemistry (Scale=50 µm).

A subepidermal diffusely grown pigmented (melanotic) neurofibroma WHO grade I was diagnosed (Figure 3 (Fig. 3)).

## Discussion

This report describes the surgical treatment of a large melanotic neurofibroma of the back in a NF1-patient. The diagnosis of the tumor subtype was suspected intraoperatively and confirmed histologically. The concept of the surgical treatment was independent of this finding. The manifestation of melanotic neurofibroma is a variant of a disease in which pigmentation disorders are used to classify and diagnose the entity (phakomatosis).

Hyperpigmentation of the skin in NF1 patients is a typical feature of the phenotype. The pigmentation disorder manifests itself both in the diagnostically café au lait spots and in a common freckling of the skin in regions that are barely exposed to sunlight. Furthermore, in many adult NF1 patients diffuse pigmentation of the skin is striking, which causes a slight gray discoloration of the skin, at least in white individuals. For these reasons, an association between the genetic alteration in NF1 and the development and differentiation of melanocytes has been suggested early [15]. 

The histogenesis of the melanotic changes in the neurofibroma is based on conjectures derived from the joint development of Schwann cells and melanocytes. The hyperpigmentation apparently results from either an atavism of Schwann cells, which as well as melanocytes are derived from the neural crest, or from atypical melanocytes that are incorporated into the tumor and produce melanin [16]. In fact, melanocytes of the skin in NF1 patients differ from normal melanocytes [12]. Immunohistochemical and ultrastructural findings support a melanotic line of differentiation in the pigmented neurofibroma [17]. On the other hand, it is known from animal experiments that changes in the NF1 gene can already occur in cells of the neural crest which will later mature to melanoblasts and alter the phenotype of the melanocytes. This experimental evidence justifies the hypothesis that somatic mutations in so-called precursor cells of neural crest, which can differentiate into both Schwann cells and melanocytes, are responsible for the development of melanotic PNF [16].

In contrast to the circumscribed differentiation disorder of the non-transformed melanocytes of the *skin* in NF1 patients, the pigmented cells in the neurofibroma belong to a diffusely infiltrative neoplasia, suggesting a different pathogenesis of these two conditions.

NF1 is one of the most common tumor predisposition syndromes in humans [18]. Furthermore, NF1 is a genetic disease that poses a relatively high risk for affected patients to develop cancer. Among the cells of origin of cancer in NF1 are those of the connective tissue [19].Therefore, increased attention to pathological changes in connective tissue is indicated in these patients. Indeed, malignant melanoma in NF1 occurs [20] although the question is not definitively clarified whether malignant melanomas arise more frequently in NF1 than in the normal population [19], [21], [22], [23]. Indeed, in a recent analysis of the causes of death of NF1 patients, MPNST are the most common malignant tumors that can lead to death, while malignant melanoma did not play a prominent role [19]. However, recent basic research and clinical studies show that somatic mutations of the NF1 gene play an essential role in the pathogenesis of *sporadic* malignant melanoma [24], [25], [26], [27]. These associations have also been reported occasionally for segmental NF1 with melanoma [28].

Melanotic neurofibroma is a rare variant of neurofibroma, which is defined by the strong pigmentation of cells within the tumor [8]. Melanotic neurofibroma has been described for both NF1 and sporadic manifestations [7]. Hyperpigmented regions were found in neurofibromas of different histological differentiation, as in diffuse, combined diffuse and plexiform, combined diffuse and intraneural epithelioid, and nonspecific growth patterns. 

Histological examination of melanotic neurofibromas has shown that neither nuclear atypia nor increased proliferation rates of the tumor cells are present [7]. Further microscopic investigations have shown that even in non-pigmented neurofibromas markedly increased pigmentation may occur that escapes the naked eye [29]. Therefore, melanotic neurofibroma is considered to be another variant of this benign tumor entity [8].

In the surgical treatment of such cases a hitherto unrecognized malignant melanoma may be suspected. However, it is typical for the pigmented neurofibroma that the melanin-containing regions of the tumor show no necrosis and macroscopically the tumor appears as typical neurofibroma except for the pigmentation.

Post-operatively our patient developed a hematoma despite the fact that the resected tissue was largely cut off from the blood circulation with deep sutures. The secondary wound healing after drainage of the hematoma was uncomplicated and led to a stable wound. Complicated wound healing of extensive diffuse plexiform neurofibroma has been reported before [30] and may be due to tumor infiltration of the wound edges.

## Conclusions

Melanotic neurofibroma is a rare variant of the typical benign neurofibroma. Careful investigation of specimen is mandatory to exclude the rare simultaneous occurrence of both malignant melanoma and neurofibroma in the cancer predisposition syndrome NF1. Wound healing may be complicated, but a permanent and stable wound healing can be expected.

## Notes

### Competing interests

The authors declare that they have no competing interests.
